# Longitudinal Associations Between Childhood Obesity and Academic Achievement: Systematic Review with Focus Group Data

**DOI:** 10.1007/s13679-017-0272-9

**Published:** 2017-07-10

**Authors:** Anne Martin, Josephine N. Booth, Sarah McGeown, Ailsa Niven, John Sproule, David H. Saunders, John J. Reilly

**Affiliations:** 1 Centre for Population Health Sciences, University of Edinburgh, Teviot Place, Edinburgh, EH8 9AG UK; 2 Institute for Education, Community and Society, Moray House School of Education, University of Edinburgh, Edinburgh, UK; 3 Physical Activity for Health Research Centre, Moray House School of Education, University of Edinburgh, Edinburgh, UK; 4 Physical Education and Health Sciences, Moray House School of Education, Institute for Sport, University of Edinburgh, Edinburgh, UK; 5 Physical Activity for Health Group, School of Psychology Sciences and Health, University of Strathclyde, Glasgow, UK

**Keywords:** Children, Adolescents, Obesity, Academic achievement, Systematic review, Longitudinal cohort studies

## Abstract

**Purpose:**

The purposes of this study were to review the evidence on longitudinal associations between child and adolescent obesity and academic achievement and to provide perceptions of adolescents with obesity and their parents on this topic.

**Recent Findings:**

Synthesis of 31 studies (from 17 cohorts) suggested that relationships between obesity and academic achievement are not well established, except for adolescent girls’ maths attainment, potentially mediated by both weight-related bullying and executive cognitive functions. Focus groups with adolescent girls with obesity confirmed experiences of psychosocial distress at school particularly during Physical Education. Adolescents perceived that obesity was not related to academic achievement directly, but by their attitude to school.

**Summary:**

Interventions are warranted to promote psychosocial wellbeing and cognitive abilities linked to academic achievement in adolescent girls with obesity. Physical Education should be a positive experience for children and adolescents with obesity.

## Introduction

The prevalence of childhood obesity is increasing in many countries with adverse societal impact [1]. Nevertheless, to date, successful efforts to tackle the problem of childhood obesity have been insufficient [1]. Further economic evaluation is required, including an improved understanding of the link between academic achievement and childhood obesity ‘strengthening the economic arguments for interventions’ [1]. A high value is placed on academic achievement by policymakers, schools, and families [2]. If obesity in childhood and adolescence is related to academic achievement, this would provide increased support for interventions aimed at preventing and treating obesity in young people.

‘Direct’ mechanisms for how childhood obesity could be associated to poorer academic achievement have been postulated [3, 4] which are associated with children’s cognitive ability [5, 6••]. ‘Indirect’ mechanisms could include obesity-related adverse physical and mental health leading to increased school absenteeism [7], obesity-related psychosocial distress (e.g. isolation, bullying) [8, 9], stigmatisation by peers and teachers [10, 11], poor sleep due to obesity-related disordered breathing [12, 13], cardio-metabolic co-morbidities [14–16], nutritional intake [17] and low levels of physical activity or fitness [18].

Two literature reviews have examined the association between overweight/obesity and academic achievement [19, 20]. Both concluded, from cross-sectional data, that children and adolescents with overweight and obesity typically perform less well in school compared to normal-weight peers. Caird et al. [19] noted a lack of longitudinal studies addressing potential mediators of the relationship between childhood obesity and academic achievement. Since the publication by Caird et al. [19] further evidence on the longitudinal relationship between obesity and academic achievement has emerged. Further, limited research has been undertaken to gain insight into the views of adolescents with obesity and their parents on the link between obesity and academic achievement. Adolescent girls with obesity tend to experience weight-related bullying in school more often which might make them more vulnerable to lower academic achievement [21]. Therefore, the aims of this study were to systematically review and critique the evidence on the longitudinal associations between childhood obesity and academic achievement and to complement the review findings with qualitative data on the perspectives of adolescent girls specifically, and their parents. The following research questions were addressed:

1. Is there evidence of a longitudinal association between childhood or adolescent obesity and academic achievement?
2. Does a change in obesity status over time influence the association with academic achievement?
3. What factors moderate or mediate the association between childhood or adolescent obesity and academic achievement?
4. How do adolescent girls with obesity and their parents perceive the link between obesity and academic achievement?

## PART 1: Methods and Results of the Systematic Review

### Methods

In January 2017, a literature search was performed in Medline, Embase, PsycINFO, Education Resource Information Centre and SportDiscus; this was restricted to English language but not publication year. The search strategy was adapted for each database and is shown for Medline (ovid) in Table 1. Reference lists and forward citations of included studies were screened. Titles and abstracts were screened independently for eligibility (AM, JNB). Full-text articles were independently screened for inclusion by AM, JNB and SM (DHS arbitrated) using the following inclusion criteria:

(i). Study design: observational prospective cohort studies
(ii). Participants: healthy 3–18-year olds (i.e. absence of conditions associated with overweight/obesity and impaired school performance)
(iii). Exposure: measures or estimates (i.e. self-reported) of body weight status as either body mass index (BMI; values, percentiles or *z* scores), body fat or waist circumference. Eligible exposures were also the BMI-derived weight status classifications overweight, obesity and overweight and obesity combined
(iv). Outcome: measures or estimates of academic achievement limited to standardised test scores, teacher- or self-reported grades of specific school subjects or average attainment during compulsory education

**Table 1:** Search strategy for ovid Medline

1. exp Obesity/ or exp Overweight/
2. (overweight or overweight or overweight).tw.
3. obes*.tw.
4. exp. Body Mass Index/
5. (body mass index or bmi).tw.
6. exp. Adiposity/
7. adipos*.tw.
8. exp. Child/
9. exp. Adolescent/
10. child*.tw.
11. (adolesc* or youth or teen*).tw.
12. young people.tw.
13. (students or pupil*).tw.
14. 8 or 9 or 10 or 11 or 12 or 13
15. exp. Education/
16. exp. Schools/
17. exp. Achievement/
18. ((school or academic* or education*) adj2 (attainment or performance or achievement* or outcome*)).tw.
19. (math* or reading or writing or science).tw.
20. 15 or 16 or 17 or 18 or 19
21. exp Prospective Studies/mt [Methods]
22. exp Longitudinal Studies/mt [Methods]
23. cohort.tw.
24. longitudinal.tw.
25. prospective.tw.
26. 1 or 2 or 3 or 4 or 5 or 6 or 7
27. exp Cohort Studies/
28. 21 or 22 or 23 or 24 or 25 or 27
29. 14 and 20 and 26 and 28
30. limit 29 to English language

Experimental studies were excluded as these are systematically reviewed elsewhere [22]; an update of the evidence from experimental studies is currently in progress.

Data were independently extracted by two reviewers and cross-checked using a tested and pre-defined data extraction template. Included studies were independently scored for methodological quality by AM and SM, disagreements were resolved through discussion. Quality assessment was scored using criteria for observational longitudinal research [23]. Quality domains included sampling and recruitment, participant characteristics, attrition, data collection methods, and data (total 19 items). Quality criteria were scored as positive, negative or ‘unclear’. If a study provided no or insufficient information, we scored the criterion at issue as ‘unclear’. Where the study referred to additional publications, we retrieved the publication to score the quality criterion. Studies were considered of high methodological quality when the percentage of items that scored positively was ≥70% [24].

A narrative evidence synthesis was performed for which individual study findings were synthesised graphically by school subjects, type of obesity measure and sex. The primary analysis considered the study quality in that studies with an overall quality score of <70%, and studies with a quality score ≥70% but with self-reported weight and height, and/or academic achievement were removed from the evidence synthesis. Where both objectively and subjectively assessed academic achievement was available, findings of objectively obtained scores were considered for the evidence synthesis. Study and population characteristics and assessment tools for academic achievement varied substantially between included studies. Although several studies utilised similar cohort studies for their analyses, combining effect sizes of the same study population would result in overestimation of the effect size [25]. Therefore, no meta-analysis was performed.

### Results

**Fig. 1:**
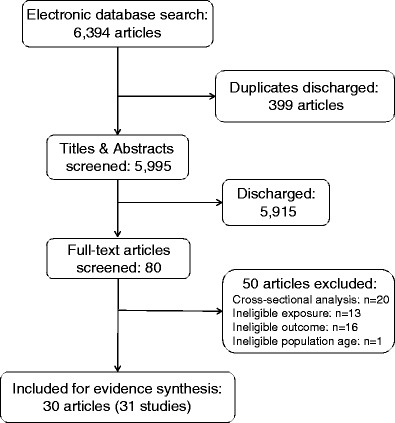
Literature search and study selection flow diagram

The systematic literature search results were summarised in Fig. 1. Eighty potentially relevant full-text articles were identified, of which 30 articles (31 studies) were included in this review.

A detailed description of population characteristics, exposure, outcomes, confounding/mediating variables and main results of the included studies is provided in Table 2. The included studies comprised 17 distinct cohort datasets, of which 11/31 studies performed secondary analysis of the Early Childhood Longitudinal Study—Kindergarten Class (ECLS-K) in the USA [26–36]. Seven studies used five other datasets from the USA [35, 37–42], and eight studies utilised existing cohort datasets from Canada [43, 44], the UK [45, 46], The Netherlands [47, 48], Australia [49], and Taiwan [50]. Five studies from Australia, Germany, Taiwan, Thailand, and Peru used primary data for their prospective analyses [51–54]. The number of participants ranged from 405 to 21,260 (median 3362 participants) and the follow-up periods ranged from 1 to 9 years (median 3 years). Twenty-four studies assessed the association between overweight and/or obesity and academic achievement whereas two studies related percentage body fat [46, 53], one study BMI *z* scores [32] and two studies BMI [40, 54], to children’s academic achievement. Measures of academic achievement were obtained from school records or standardised tests by 25 studies and five studies relied on teacher or self-reported academic attainment [37, 42, 44, 52, 55].

**Table 2:** Characteristics of included studies reporting on the longitudinal relationship between child and adolescent obesity and academic achievement

Reference (quality score)	Sample characteristics	Exposure: measure of body weight status	Outcome: measure of academic achievement	Main results	Confounders
Afzal et al. 2015 (58%)	Location: USA Cohort: NLSY *N* = 2672 (cohort 1), 1991 (cohort 2) Age: 2–8 years (baseline), 8–16 years (follow-up) Sex: 50.2% (f, cohort 1), 48.8% (f, cohort 2)	BMI^a^ Persistent obesity^e^ Developed obesity^e^ Grew out of obesity^e^	Maths, reading Peabody Individual Achievement Test (PIAT)	• n.s. association between change in OB and attainment in boys and girls	Sex, maternal education, maternal ethnicity, maternal obesity, poverty level, Home Observation Measurement of the Environment, child’s height
Bisset et al. 2012 (85%)	Location: Canada Cohort: Quebec Longitudinal Study of Child Development *N* = 1959 Age: 4–7 years (baseline), 8.2 years (follow-up) Sex: 49.7% (f)	BMI^a^ Overweight^c^	Average of reading, math, writing Teacher report	• n.s. association between OW and academic achievement (crude and adjusted model)	Gender, breastfeeding duration, whether low birth weight, socio-familial adversity index, cognitive abilities aged 3y and 7y, internalising/externalising behaviour problems
Black et al. 2015 (89%)	Location: Australia Cohort: Longitudinal Study of Australian Children *N* = 7225 Age: 4–5 years (baseline), 12–13 years (follow-up) Sex: 49% (f)	BMI^a^ *z* score Overweight^c^ Obesity^c^	Maths, literacy National Assessment Program—Literacy and Numeracy	• n.s association between OW and maths and literacy in boys and girls • OB among boys is associated with a 0.24 SD (SE (0.081) ↓ maths score and a 0.23 SD (SE 0.089) ↓ literacy score • Significant negative association between BMI *z* scores and maths (−0.068 SD (SE 0.024)) and literacy (−0.055SD (SE 0.023)) scores in boys • Significantly ↓ maths and literacy scores in girls with OB and for BMI *z* scores; n.s. association after controlling for cognitive abilities	Child’s age in months, age squared, region of residence, number of younger/older siblings, single-parent family, ethnicity, mother and father’s education level, household income quintiles and mother’s employment status, school type, teacher’s years of experience, low (<2500 g) birth weight, whether breast-fed at 6 months of age, mother’s smoking status while pregnant, maternal mother’s age at Birth, home environment index, cognitive ability
Booth et al. 2014 (95%)	Location: UK Cohort: ALSPAC *N* = 4260 Age: 11 years (baseline), 13 and 16 years (follow-up) Sex: 55% (f)	BMI^a^ *z* score Overweight^d^ Obese^d^ Developed obesity^d^ Persistent obesity^d^ Became healthy weight^d^	English, maths, science Standardised National Exams (Key Stage 2, 3, 4)	• Significantly ↓ English, maths and Science grades in girls with OB at 13 and 16 years • ↓ English grades in persistent OW/OB girls and OW➔OB girls • n.s. association for boys with OW/OB and girls with OW • n.s. association for OW−/+, OB−/+, OW/OB +/−	Age, birth weight, gestation; age of mother at delivery, mother’s oily fish intake during pregnancy at 32 weeks gestation, maternal smoking in the first 3 onths of pregnancy; pubertal status, menarche status, maternal education, maternal occupational status, MVPA/week, depressive symptoms, full IQ, BMI *z* score at age 16 yrs
Capogrossi et al. 2013 (74%)	Location: USA Cohort: ECLS-K *N* = 21,260 Age: 1st grade (baseline), 8th grade (follow-up) Sex: 49% (f)	BMI^a^ *z* score	Maths, Reading ECLS-K test based on Woodcock-McGrew-Werder Mini-Battery of Achievement	• Significantly negative association between reading scores in boys in 5th grade and BMI *z* scores • n.s. association in girls • n.s. association in boys for reading in 8th grade and maths in 5th grade • Positive association between BMI *z* scores and reading in boys 8th grade	Baseline BMI *z* score, birth weight, age, baseline attainment score, previous attainment score, urbanicity, whether parents are married, number of siblings, household income, how involved the parent is in the child’s school, how often the parent helps the child with homework, how often the child has changed schools, whether the child has a consistent bedtime, the number of students enrolled in the school, whether the school is Title 1, number of years the teacher has taught and whether the teacher has a Master’s degree
Carter et al. 2010 (84%)	Location: Canada Cohort: National Longitudinal Survey of Children and Youth *N* = 2582 Age: 2–5 years (baseline), 8–11 years (follow-up) Sex: 48.0% (f)	BMI^b^ Persistent obesity^c^ Developed obesity^c^ Grew out of obesity^c^	Maths Mathematics Computation Test of CAT/2, shortened version (IRT)	• Significantly ↑ maths scores in O+/− students than O- • n.s. association between O+ and O−/+ and math attainment	Age, gender, ethnicity, sleep, physical activity, chronic condition status, birth weight, household income, family structure; maternal education, working status, age at birth of child, smoking status, degree of positive parenting
Chen et al. 2012 (68%)	Location: Taiwan Cohort: primary study *N* = 409 Age: 6 years (baseline), 12 years (follow-up) Sex: 48.4% (f)	BMI^a^ Persistent obesity^c^ Developed obesity^c^	Average score of language, maths, science, social studies School records	• n.s. negative association O+ and O−/+ and average attainment	School absenteeism, IQ (Raven’s Colored Progressive Matrices), gender, parental education, number of siblings, family structure
Cueto 2005 (84%)	Location: Peru Cohort: primary study *N* = 438 Age: 12 years (baseline), 14y (follow-up) Sex: 51% (f)	BMI^a^	Maths, reading Local attainment test	• n.s. negative association between BMI and maths and reading	Student dropped out, baseline achievement, grade in school
Crosnoe & Muller 2004 (79%)	Location: USA Cohort: Add Health *N* = 11,658 Age: 15 years (baseline), 16 years (follow-up) Sex: 51% (f)	BMI^b^ Overweight^e^	Grade point average of maths, science, English, and social studies Self-reported A-F	• Significantly ↓ average attainment in OW students compared to healthy weight peers	Gender, age, ethnicity, parental education, family structure, prior attainment, athletic activities, educational aspiration, school attendance, homework efforts, participation in non-athletic activities, romantic activities, school SES, minority representation, school mean BMI
Datar et al. 2004 (84%)	Location: USA Cohort: ECLS-K *N* = 11,192 Age: Kindergarten (baseline), 1st grade (follow-up) Sex: 49.93(f)	BMI^a^ Obesity^e^	Math, reading ECLS-K test based on Woodcock-McGrew-Werder Mini-Battery of Achievement	• n.s. negative association between OB and maths and reading attainment in boys and girls	Hours/day watches television or videos, days/week child exercises for ≥20 min, number of activities that parent participates in with child at least once a week, birth weight, baseline test score, ethnicity, mother’s education, family income, urbanicity
Datar & Sturm 2006 (53%)	Location: USA Cohort: ECLS-K *N* = 7090 Age: kindergarten (baseline), 3rd grade (follow-up) Sex:51 (f)	BMI^a^ Persistent obesity^e^ Developed obesity^e^	Maths, reading ECLS-K test based on Woodcock-McGrew-Werder Mini-Battery of Achievement	• Significantly ↓ maths and reading scores in O−/+ girls compared to O- in girls • n.s. association for O−/+ boys and O+ boys and girls	Gender, age in months in spring of third grade, race/ethnicity, birth weight, annual family income, mother’s education, single-parent household, maternal depression scores, parent–child interaction, hours of television watching, parent-reported physical activity, amount of physical education, school characteristics such as enrolment, percent minority, and urbanicity
Gable et al. 2008 (56%)	Location: Cohort: ECLS-K *N* = 8000 Age: Kindergarten (baseline), 1st and 3rd grade (follow-up) Sex:52.0%	BMI^a^ Persistent obesity^e^ Developed obesity^e^	Reading, Maths ECLS-K test based on Woodcock-McGrew-Werder Mini-Battery of Achievement	• Significantly ↓ maths and reading scores in O−/+ compared to never-obese children • n.s. association between O+ and maths and reading	Ethnicity and SES
Gable et al. 2012 (72%)	Location: USA Cohort: ECLS-K *N* = 6250 Age: Kindergarten (baseline), 3rd and 5th grade (follow-up) Sex: 52.0% (f)	BMI^a^ Persistent obesity^e^ Developed obesity^e^	Maths ECLS-K test based on Woodcock-McGrew-Werder Mini-Battery of Achievement	• Significantly ↓ maths scores in O+ and O−/+ boys and girls compared to O-	Child age at study enrolment, ethnicity, maternal education, maternal employment status, parental psychological well-being, parent expectations of child educational achievement, household income, family structure, child’s interpersonal skills, internalising behaviour
Kenney et al. 2015 (88%)	Location: USA Cohort: ECLS-K *N* = 3362 Age: 5th grade (baseline), 8th grade (follow-up) Sex: 49.4% (f)	BMI^a^ BMI *z* scores^e^ Overweight^e^ Obesity^e^	Maths, reading ECLS-K test based on Woodcock-McGrew-Werder Mini-Battery of Achievement Teacher reported: Academic Rating Scale: Poor (1) – Outstanding (5)	• n.s. negative association between change BMI *z* scores and standardised maths and reading scores in boys and girls • One-unit ↑ in BMI *z* score significantly associated with a 0.12 SD ↓ in teacher ratings of girls’ reading ability (95%CI:- 0.23,-0.01) • One-unit ↑ in BMI *z* score significantly associated with a 0.30 SD ↓ in teacher ratings of boy’s maths ability (95%CI:-0.43,-0.17)	Race/ethnicity, SES (annual family income and highest parental education), physical activity, television watching, maternal depression, overall child health, family structure, parent–child interaction, parental disciplinary behaviours
Kranjac 2015 (89%)	Location: USA Cohort: ECLS-K *N* = 5072 Age: 5.7 years (baseline), 14.1 years (follow-up) Sex: 44.6% (f)	BMI^a^ Overweight^c^ Obesity^c^ Developed overweight^e^ Developed obesity^e^	Maths ECLS-K test based on Woodcock-McGrew-Werder Mini-Battery of Achievement	• Significantly ↓ maths score (5.77 points, SE 0.07) in OW • Significantly ↓ maths score (7.97 points, SE0.69) in OB • Effect of obesity on maths achievement is stronger as children age • Maths trajectories of children with OW with high levels of self-efficacy ↑by 3.62 points more than children with OW with low levels of self-efficacy (*p* < 0.005) • n.s mediating effect of self-efficacy in children with OB	Gender, ethnicity, parental education, self-efficacy
Li & O’Connelly 2012 (67%)	Location: USA Cohort: ECLS-K *N* = 6178 Age: 5.7 years (baseline), 11.2 years (follow-up) Sex: 50.0% (f)	BMI^a^ Persistent obesity^e^	Maths, reading ECLS-K test based on Woodcock-McGrew-Werder Mini-Battery of Achievement	• n.s. negative association between OB and maths/reading	Gender, ethnicity, SES, parental education, mother’s education, single-parent household, primary language at home, school type, school location, percentage minority
Lu et al. 2014 (68%)	Location: Taiwan Cohort: Taiwan Education Panel Survey *N* = 8690 Age: 7th grade (baseline), 9th grade (follow-up) Sex: 50.7 (f)	BMI Overweight^g^	Average attainment Comprehensive cognitive ability (CCA) scores	• Significantly ↓ average scores in boys (−1.04, SE 0.57) and girls (−1.66, SE 0.78) with OW	CCA score in the 7th grade, gender, own expected education level and ability education level, family income, parents’ education level, marital status, school location, school type
Manes 2015 (84%)	Location: USA Cohort: NICHHD Study of Early Child Care and Youth Development *N* = 915 Age: 9 years (baseline), 15 years (follow-up) Sex:50.8% (f)	BMI^a^	Maths, reading Woodcock-Johnson Psycho- Educational Battery – Revised (WJ-R)	• Significant association between ↑BMI and ↓ reading and maths attainment • n.s. association between BMI and reading and maths attainment after controlling for cognitive abilities (executive function, concentration)	SES, gender, executive function, concentration, internalising behaviour
Mo-Suwan et al. 1999 (89%)	Location: Hat Yai municipality, southern Thailand Cohort: primary study *N* = 2252 Age: 3rd–6th grade (baseline), 7th–9th grade (follow-up) Sex: 53.4% (f)	BMI^a^ Persistent overweight^f^ Developed overweight^f^ Grew out of overweight^f^	Grade Point Average in maths and Thai Language Teacher reported	• Significantly ↓ average scores in OW−/+ compared to OW- • n.s. association between OW+ and O+/− and attainment	Age, gender, school, grade
Murasko et al. 2015 (78%)	Location: USA Cohort: ECLS-B and ECLS-K *N* = 9950 (ECLS-B); *N* = 18,820 (ECLS-K) Age: B = 16.83 months (baseline), 57.75 months (follow-up); ECLS-K = 5.7 years (baseline), 14.1 years (follow-up) Sex: B = 48.8% (f); K = 48.7% (f)	BMI^e^ Overweight^e^ Obesity^e^	Maths, reading ECLS-B test items taken from: PreLAS 2000; Peabody picture vocabulary; Preschool Comprehensive Test of Phonological and Print Processing; Test of Early Mathematical Ability-3 ECLS-K test based on Woodcock-McGrew-Werder Mini-Battery of Achievement	ECLS-B: • n.s. association between maths and reading and boys and girls with OW and OB ECLS-K: • Significantly ↓ maths scores in girls (−0.74, SE 0.17); n.s. for girls with OW, • n.s. association between reading and girls with OW and OB • n.s. association between maths and reading in boys with OW and OB	Age, gender, ethnicity, birthweight, household size, presence of mother (resident), maternal age, resident father, paternal age, parents educational level, household income
Palermo & Dowd 2012 (89%)	Location: USA Cohort: Child Development supplement of Panel Study of Income Dynamics *N* = 2820 Age: 8.6 (baseline); 11.69 (1st follow-up) and 13.98 (2nd follow-up) Sex: 50% (f)	BMI^a^ Overweight^e^ Obesity^e^	Reading Broad reading score of the Woodcock-Johnson Revised Tests of Achievement (WJ-R)	• n.s. association between reading in boys and girls with OW and OB	Race/ethnicity, gender, age, parental education, household income
Roberts & Hao 2013 (78%)	Location: USA Cohort: Teen Health 2000 study *N* = 3134 Age: 11–17 years (baseline), 12–18 years (follow-up) Sex: not reported	BMI^a^ Overweight^e^ Obesity^e^	Average attainment (good vs poor) Teacher reported	• n.s reduced odds of poor school performance in OW and OB boys and girls	Age, gender, family income, prior academic performance.
Ruijsbroek et al. 2015 (79%)	Location: The Netherlands Cohort: PIAMA *N* = 1531 Age: 3–5 years (baseline), 6–8 years and11 years (follow-up) Sex: 51% (f)	BMI^b^ Overweight^c^ Persistent overweight^c^ Developed overweight^c^ Grew out of overweight^c^	Average of Spelling, maths, study skills and world studies Cito test z scores	• Cito test scores were significantly ↓ children with OW (−0.04 *z* score (95% CI −0.07; 0.00) per year with overweight) •n.s. association between attainment and OW+, OW−/+ and OW+/−	SES (maternal education level), sex
Sabia & Rees 2015 (63%)	Location: USA Cohort: Add Health *N* = 11,822 Age: 7th grade (baseline), end of high school (follow-up) Sex: 52.2% (f)	BMI^b^ at baseline BMI^a^ at follow-up Overweight^e^ Obesity^e^	Cumulative high school grade point average (GPA) School records	• Significantly ↓ GPA in girls with OW (−0.123 points, SE 0.03) and OB (−0.289 points, SE 0.04) • Significantly ↓ GPA in boys with OB (−0.071, SE 0.04), n.s. association in boys with OW	Parental education, household income, parental marital status, child’s cognitive ability, race, religiosity, age, number of biological siblings, birth order, percentile height-for-age, pubertal development, disability status, and attractiveness of the child’s personality and grooming, self-esteem, depression,
Suchert et al. 2016 (83%)	Location: Germany Cohort: primary cohort *N* = 1011 Age: 14.1 years (baseline), 15.0 years (follow up) Sex: not reported	BMI^a^ Overweight^f/^ Obesity^f^ Developed overweight Persistent overweight Grew out of overweight	Average grade of maths and German Self- reported grades (1–6, lower indicates better)	• Significantly ↓ attainment in OW−/+ (−0.18 grades, 95%CI −0.35; −0.01) • n.s. association between academic attainment and OW and OB • n.s. association between academic attainment and OW+, OW+/−	Sex, age, type of school students attend
Telford et al. 2012 (71%)	Location: Australia Cohort: primary cohort *N* = 757 Age: 8.5 years (baseline), 10.5 years (follow-up) Sex: 47.0% (f)	Body Fat (DEXA)	Maths, reading, writing Local government education authority	• n.s. association between %BF and attainment	SES
Veldwijk et al. 2012 (83%)	Location: The Netherlands Cohort: PIAMA *N* = 1543 Age: 8 years (baseline), 12 years (follow-up) Sex: 51% (f)	BMI^a^ at 8y BMI^b^ at 12y Overweight^c^ Persistent overweight^c^ Developed overweight^c^	Average of spelling, maths, study skills and world studies Cito test z scores	• n.s. negative association OW, OW+ and OW−/+ and average attainment	Gender, maternal smoking, maternal age at birth, breastfeeding duration, birth weight, parental education, lifestyle factors (physical activity, screen time, breakfast skipping), child’s psychological health, being bullied, school absenteeism due to illness
von Hinke Kessler Scholder et al. 2012 (82%)	Location: UK Cohort: ALSPAC *N* = 3001 Age: 9 and 11 years (baseline), 11 and 14 years (follow-up) Sex: 51% (f)	Fat mass (DEXA) at age 9 and 11 adjusted for height, height ^2^, gender, age	Average of English, maths, science National exams (Key Stage 2 and 3)	• Significantly negative association between fat mass at 11 years and average scores at age 14 years • n.s association between BF at 9 years and average scores at age 11 years	Birth weight, number of siblings, age, family income, mother’s education, whether mother smoked or drank alcohol during pregnancy, mother’s mental health, maternal age at birth, length of breast feeding, mother’s parents education, raised by natural father, family’s social class, parental employment status, parental involvement in child development, area deprivation
Wendt 2009 (95%)	Location: U.S.A Cohort: ECLS-K *N* = 12,719 Age: Kindergarten (baseline), 3rd grade (follow-up) Sex: 49% (f)	BMI^a^ Persistent obesity^e^ Developed obesity^e^	Reading, maths ECLS-K test based on Woodcock-McGrew-Werder Mini-Battery of Achievement	• Significantly ↓ maths scores in OW, O+ and O−/+ boys and girls • n.s association between O+ and O−/+ and reading in boys and girls	Birth weight, number of school changes, frequency student reads by him/herself /week, learning problems, bedtime, TV viewing, hours of non-parental care/ week, ethnicity, gender, grade level, parent’s involvement with student’s school activities, student lives with both biological parents, number of places a student lives during the last year of interview time, parents’ expectation for student’s schooling, number of siblings, SES, household has computer for student’s use, teacher enjoys teaching, teacher’s years of teaching, teacher’s degree, teacher is White, private school, % of student in school tested at or above grade level on national standardised, school experiences problems of teacher’s turn-over rates, School’s size, % of minority students, school location, security problems
Zavodny et al. 2013 (78%)	Location: USA Cohort: ECLS-K *N* = 18,820 Age: 1st grade (baseline), 8th grade (follow-up) Sex: 49% (f)	BMI^a^ Overweight^e^ Obesity^e^	Language, reading, maths, science ECLS-K test based on Woodcock-McGrew-Werder Mini-Battery of Achievement	• Significantly ↓ maths and reading/language scores in OB students compared to healthy weight peers • n.s association between OB and science • n.s association between OW and maths, reading and science	Sex, race/ethnicity, birth weight, foreign-born status, hours of television watched/week; number of siblings, SES, school region, urban/ suburban/rural, public/private non-religious/ Catholic/ other religious, percent minority students, percent of students receiving free lunch; teachers’ age, years of teaching, teachers’ education, indicator variable for the teacher and child being the same race/ethnicity

*f* female, *O−* never with obesity, *O−/+* developed obesity, *O+/−* grew out of obesity, *O+* persistent obesity, *OW+* persistent overweight, *OW−/+* developed overweight, *OW* overweight, *NOW* non-overweight, *OB* obesity, *BMI* Body mass index, *BF* body fat, *f* German Reference Population, *IOTF* International Obesity Task Force, *CAT/2* The Standardised Canadian Achievement Test: 2nd edition, *IRT* item response theory, *Cito test* Central Institute for Test Development test, *IQ* intelligence quotient, *NLSY* National Longitudinal Survey of Youth, *ECLS-B* Early Childhood Longitudinal Study—Birth Cohort, *ECLS-K* Early Childhood Longitudinal Study-Kindergarten Cohort, *ALSPAC* Avon Longitudinal Study of Parents and Children, *Add Health* National Longitudinal Study of Adolescent Health, *NICHHD* National Institute of Child Health and Human Development, *PIAMA* Prevention and Incidence of Asthma and Mite Allergy, *n.s.* non-significant (*p* > 0.05)

^a^Objectively reported weight and height

^b^Self/parental reported weight and height

^c^Cut-offs based on IOTF classification

^d^UK 1990 reference population

^e^Centre for Disease Control and Prevention growth reference charts

^f^US National Health And Nutrition Examination Survey (wave 1) reference

^g^Department of Health, Executive Yuan in Taiwan

**Fig. 2:**
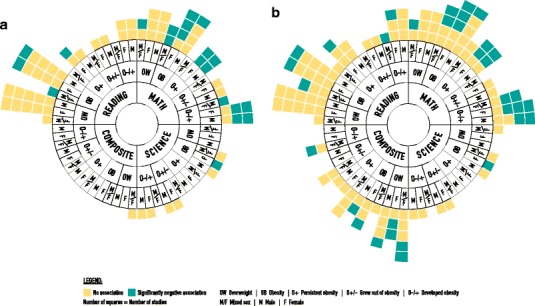
Evidence synthesis on the longitudinal association between child and adolescent overweight and obesity and academic achievement. **a** Primary analysis with 15 high-quality studies. **b** 24 high- and low-quality studies. Each study may be represented in multiple outcomes and weight categories

Sixteen studies assessed the association between overweight or obesity at one time point and academic achievement later in life (Fig. 2a). Of those, eight studies were excluded from the primary analysis due to methodological shortcomings (Fig. 2b). Fourteen studies using data from eight cohorts assessed the association between change in obesity status and academic achievement (Fig. 2b). However, only four studies (two cohorts) were of high methodological quality and so included in the primary analysis (Fig. 2a). Change in obesity status was classified as persistent obesity, development of obesity, and ‘growing out’ of obesity (change from obesity to overweight or healthy weight).

All included studies adjusted for a number of confounding variables known to be associated with both obesity and academic achievement such as measures of socioeconomic status (e.g. parental education, family income). The quality scoring of each individual study ranged from 53% [26] to 95% [30, 45]. The level of agreement for the quality scoring between the reviewers (AM, SM) was 96% (*k* = 0.91). In 24/30 studies, methodological quality was scored high (≥70%).

### Association Between Overweight or Obesity and Academic Achievement

Figures 2a, and b shows the graphically synthesised data of the included studies on the association between child and adolescent overweight and obesity and academic achievement, namely reading/language, maths, science and a combined average of school subjects. Table 1 also summarises the results for each of the included studies.

#### Overweight and Academic Achievement

Five high-quality studies (four articles) assessed the link between overweight and maths achievement with one study [34] suggesting significantly lower maths scores in adolescents with overweight at 14 years compared to healthy weight peers (*n* = 5072). Four studies reported that there was no evidence of an association between overweight and maths achievement [31, 35, 49].

In terms of reading achievement, six high-quality studies (five articles) consistently concluded that there is no evidence of a significant negative association between overweight and reading achievement [31, 35, 41, 45, 49].

Overweight-related associations with science achievement was assessed in one study (*n* = 18,820) which indicated no significant relationship between overweight at age 7 years and science achievement at 14 years [31].

Eight studies assessed the association between overweight and average achievement score of multiple school subjects [37, 38, 42, 44, 47, 48, 50, 55]. All but one study [47] showed methodological limitations and so were removed from the primary analysis (Fig. 2a). The study indicated that there is no significant association between children aged 8 years with overweight and average achievement at 12 years (*n* = 1543).

#### Obesity and Academic Achievement

Ten articles assessed the association between obesity at baseline and academic achievement at follow-up [27, 31, 35, 36, 38, 39, 42, 45, 47, 49].

Based on three high-quality studies using data from three distinct cohorts (*n* = 8641), there was consistent evidence for significantly lower maths scores at age 13–14 years in girls with obesity compared to healthy-weight peers [35, 45, 49]. This finding was not evident in preschool-aged girls with obesity [35]. Two studies, provided data jointly for girls and boys from the ECLS-K cohort, and suggested a significantly negative association between obesity at age 5–7 years and maths achievement at age 14 years [31, 34]. For boys with obesity, three of the four cohort studies did not find a significant association [35, 45].

Six high-quality studies (four articles) provided evidence on reading achievement in children with obesity compared to children with healthy weight (Fig. 2a). One study considered boys and girls as one study sample and found that students with obesity at age 7 years performed less well in reading and language achievement at age 14 compared to peers with healthy weight [31]. Where studies distinguished between reading achievement of boys and girls, 4/5 studies suggested no association in both sexes (Table 2).

There was no evidence of an association between obesity and science achievement when girls and boys were analysed as one study sample [31], whereas stratified analysis for gender suggested lower science scores in girls with obesity but not in boys at age 13 and 16 years [45].

### Associations Between Change in Obesity Status and Academic Achievement

#### Persistent Obesity and Academic Achievement

Using the same cohort (ECLS-K), two studies reported significantly lower maths achievement scores in girls and boys aged 9 and 11 with persistent obesity [29, 30].

Data from two high-quality studies (two cohorts) on reading achievement were conflicting for girls, with one study indicating a significantly negative association at age 13 and 16 years [45] and one study indicating no significant association at age 9 years [30]. The latter study also indicated no evidence of an association between boys with persistent obesity and reading achievement (Table 2).

No high-quality data were available for science scores and combined average scores of multiple school subjects (Fig. 2b).

#### Development of Obesity and Academic Achievement

There was no evidence of an association between developing obesity and reading [30, 45] and science [45] achievement in boys and girls.

Developing obesity was not significantly associated with lower maths scores in male and female adolescents aged 13 and 16 years [45] but was in younger children aged 9 and 11 years [29, 30]. When combining data of boys and girls and using a longer follow-up duration up to age 14 years, Kranjac (2015) confirmed a negative association between adolescents who developed obesity over time and maths achievement, compared to peers that maintained a healthy weight [34].

Data from high-quality studies were lacking for combined average school subject achievement.

#### ‘Growing Out’ of Obesity and Academic Achievement

Based on a single high-quality study [45], there was no evidence of an association between growing out of obesity and reading/language, maths and science achievement (Table 2). No data were available for combined school subject achievement.

### Moderating or Mediating Factors

The present systematic literature review and data synthesis of high-quality studies indicated that the association between childhood obesity and academic achievement varied by sex, age and school subject. A significantly negative association between obesity and maths achievemement was evident in adolescent girls, while the evidence suggested no association with math achievement in younger girls with obesity and in boys with obesity in general.

Out of the 30 included studies, six studies performed mediation analyses to identify the role of plausible factors that potentially mediate the relationship between child/adolescent obesity and academic achievement. The following mediating factors were assessed:

#### Cognitive Abilities

Manes (2015) concluded that childhood obesity at age 9 years indirectly predicted academic achievement at age 15 years through the cognitive processes involving executive functioning and concentration [40]. In contrast, Booth et al. (2014) suggested no mediating role of full-scale intelligence quotient between obesity at age 11 years and academic achievement at age 16 years [45].

#### Age of Menarche

Booth et al. also found no mediating effect of age of menarche [45].

#### Physical Health

One study indicated that self-reported health problems influencing performance at school did not mediate the association between obesity age 8 years and academic achievement age 12 years [47].

#### Internalising Behaviour (Including Anxiety, Self-Esteem and Depressive Symptoms)

Teacher-rated internalising behaviour was found to have a significant mediating effect between persistent obesity from preschool age and maths achievement in boys and girls aged 9 and 11 years [29]. However, Manes (2015) reported that internalising symptoms assessed using a standardised inventory did not mediate the association between obesity and maths and reading achievement [40]. This finding was supported by four other studies, which assessed the mediating role of depression [38, 45, 47, 55].

#### Self-Efficacy

Two studies consistently concluded that the association between child and adolescent obesity and academic achievement at age 14 is not mediated by general self-efficacy [34, 55].

#### Psychosocial Factors

Psychosocial distress in the form of being bullied [47] and teacher-rated interpersonal skills [29] had significant mediating effects between weight status at age 7–8 and academic achievement in girls age 11–12 years.

In summary, the current evidence suggested that cognitive processes involving executive functions and psychosocial factors might mediate the association between obesity and academic achievement.

## PART 2: Methods and Results of the Qualitative Research

This preliminary exploratory qualitative study was complementary to the quantitative data from the systematic review providing more in-depth insight of a few adolescent girls, and providing them with a voice that allows greater understanding of the relationship between obesity and academic achievement [35, 45, 49].

### Methods

Following completion of a weight management programme (Get Going NHS Lothian: www.nhslothian.scot.nhs.uk/getgoing/) in Scotland, four obese adolescent girls (aged 12–15 years, mean body mass index 99.6th percentile relative to 1990 UK reference) and one of their respective parent/guardians (i.e. 4 dyads in total) participated in separate focus groups of 60 to 90 min (i.e. two focus groups). Focus groups were led by AM, included semi-structured open-ended questions and were audio recorded and subsequently transcribed verbatim using NVivo10 [56]. Data from adolescents and parents/guardians were analysed separately following an inductive thematic analysis [57]. The text was coded and similar codes were clustered into hierarchical themes. Trustworthiness of the analysis was enhanced through independent coding (AM, AN) and member checking. This study was approved by the University of Edinburgh and the National Health Service South East Scotland Research Ethics Committee.

### Results

From the focus groups, it was evident that, despite negative body weight-related experiences in school, the girls had a generally positive attitude to education and school. They said that they do very well in school and usually outperform healthy-weight classmates. The only subject the girls mentioned that they were not good at was Physical Education (PE) and this was perceived to be related to the girls’ body weight. Parents/guardians and adolescents perceived that academic achievement is not necessarily related to body weight per se; academic achievement depends on pupils’ attitude towards learning and efforts put into school work. However, both adolescents and parents/guardians believed that the reason for the girls’ good academic achievement is that they were less distracted from school work due to the lack of friends and absence of good peer relationships. The girls said that having friends in class can lead to girls spending more time talking to their friends rather than concentrating and taking school seriously.

Additional themes emerged on female adolescents’ experiences in the school environment that were directly influenced by their body weight status. Both adolescent girls and parents/guardians reported negative psychological consequences of obesity, for example, low self-confidence and unhappiness due to body weight. Social consequences were also highlighted, for example, the girls reported difficulties being accepted and understood by healthy-weight peers in school, and were felt to be lacking friends. From both adolescents’ and parents/guardians’ responses, it emerged that the girls experienced rejection by peers in school, negative body weight-related comments, classmates laughing and gossiping about them and that at times they feel isolated and ignored. The girls viewed having a higher body weight than other teenagers in school as a disadvantage during PE, and for getting appropriately sized clothes for school (lab coats, school uniform, PE kits). PE was reported to be an environment where the girls were strongly exposed to body weight-related teasing. Some girls also felt ignored and excluded from activities by PE teachers. In contrast, classroom teachers were perceived as non-judgmental towards the increased body weight. Eating healthily in school and receiving support from classmates to do so was described as difficult and sometimes even a reason for being isolated from peers.

## Discussion

This paper builds on previous reviews on childhood obesity and its association with academic achievement [19, 20], specifically focusing on longitudinal studies. It also provides insight into mediators or moderators, and perceptions of adolescent girls with obesity and their families on the obesity-academic achievement association. In summary, the systematic evidence synthesis showed that obesity is negatively associated with adolescent girls’ maths achievement. There is some evidence that this negative association is mediated by psychosocial experiences such as body weight-related bullying and participants’ cognitive abilities involving executive functions. However, discrepancies arise for other subjects and age groups where there was less convincing evidence of associations. The role of change in obesity status over time on academic achievement is less well investigated in the majority of existing literature.

Studies in younger children and pre-adolescents generally indicated no association between obesity and academic achievement. This finding is plausible when taking into consideration the developmental trajectories of cognitive abilities related to academic achievement, social functioning and emotional control such as executive function [58, 59] (i.e. reasoning, working memory, cognitive flexibility, inhibition). These cognitive abilities begin developing in infancy but develop steeply from the age of 6 years, develop throughout adolescence and reach adult levels at about age 20 [59–61]. It is suggested that puberty influences neural reorganisation in the prefrontal cortex, the brain area linked to executive functions [62]. Therefore, obesity-related deficits in academic achievement might manifest only during adolescence [58].

Working memory, reasoning, inhibition and cognitive flexibility are strongly associated with maths achievement [63] and have been shown to be impaired in children and adolescents with obesity [6••]. Although maths and reading share multiple cognitive processes [64], for non-verbal maths tasks, different brain regions are implicated [65]. The brain regions invoked during non-verbal maths tasks are also areas (prefrontal cortex, hippocampus) which have been suggested to be associated with obesity and energy-balance related behaviours [66–67, 68••]. This might explain why obesity in childhood and adolescence appeared to be negatively associated with maths but not reading/language achievement. Further research to understand these mechanisms is warranted.

The negative association between obesity and academic achievement in girls, but to a lesser extent in boys, could be attributed to the fact that obese girls face more incidences of body weight-related stigmatisation [21] and are more likely to be distressed by teasing/bullying than boys [69, 70]. Weight-based teasing was shown to be linked to lower academic achievement [70].

Consistent with the published literature [71–75] were the experiences of participants from our focus group study who experienced social rejection, difficulties in making friends and stigmatisation, although not from teachers. Stigmatisation and teasing was particularly evident in PE, and consistent with previous research [67, 69], the girls reported that their body weight stopped them performing well in this environment. Although, it appears body weight did influence the school experience for our participants, they did not feel that their body weight directly influenced academic achievement of other school subjects specifically, but instead highlighted the importance of attitude to school. Similar to previous research [65], participants reported focusing on education rather than social relationships with peers, perhaps as a coping mechanism to avoid obesity-related bullying and peer rejection.

### Strengths and Limitations

Strengths of this work include the use of rigorous systematic review methodology and a focus on longitudinal data to provide a more nuanced insight into the association between obesity and academic achievement. The ability to distinguish between obesity and overweight as exposure variables was also a strength. Adding focus group data provided useful preliminary insight into the perceptions of adolescent girls with obesity and their parents to complement and advance the observational literature.

However, some limitations are notable. Included studies were in English language only; thus, we might have missed relevant studies published in non-English language. Focus group findings are limited in their generalisability given the low number of participants (saturation of themes potentially not reached) and the fact that all those who participated had recently completed a weight management programme (non-participation and non-completion might influence perceptions).

### Implications for Research and Practice

The current evidence on the association between obesity and maths attainment is available primarily from North American and European cohorts and entirely from high-income countries. Further research is needed to establish if an obesity-related deficit in academic achievement is evident in children and adolescents from middle-low-income countries. Given the current steep rise in the prevalence of childhood obesity in these countries [1], if there is an association between obesity and academic achievement in these contexts, the subsequent economic impact of obesity-related deficits may have even more implications for economic growth of middle-low-income countries and for human capital. Prospective cohort studies indicated that adolescent obesity is negatively associated with years of schooling [76, 77], school completion [78], enrolment in higher education [79, 80], income [76, 77, 81] and employment status [82]. The economic argument for the implementation of effective childhood obesity prevention and treatment programmes could therefore be substantial.

The current evidence is also limited on the impact of mediating factors. None of the included longitudinal studies evaluated the mediating effects of sleep deprivation, physical activity levels, type of physical activity, diet/nutritional status and co-morbidities, despite the literature suggesting an important role of these factors in the causal pathway between childhood obesity and academic achievement [12, 13, 15–17, 83]. However, most recently (after our literature search was performed), new findings were published on the link between meeting lifestyle-behaviour recommendations at age 11 years and academic achievement at age 12 years (*n* = 4253). Researchers concluded that overweight or obesity was not associated with maths or reading achievement expectation but academic achievement was associated with meeting dietary, sleep and screen time recommendations [84]. Future longitudinal studies should include mediation analysis of those factors to contribute to understanding the underlying mechanisms of a negative association between adolescent obesity and academic achievement. This in turn will help to identify the most promising intervention strategy for promoting educational outcomes. In addition, the ability to identify a factor as a mediator depends on the reliability and validity of the measure and so researchers should avoid utilising crude measurement methods.

Academic achievement might be influenced by a teacher-bias towards obesity, in that children and adolescents are perceived as having poorer reasoning, social, physical and cooperation skills which impact on academic achievement [10, 11, 33, 85•]. However, the empirical evidence is inconsistent [42, 44, 48, 52]; thus, further research to provide empirical tests of this perspective is warranted. Nonetheless, there is convincing evidence on limited educational opportunities when PE teachers hold a biased perception about the abilities of children with obesity [86–88]. Our focus group data also indicated that during PE, the girls felt ignored and not supported by the teachers. This finding highlights the importance of promoting positive PE experiences among adolescent girls with obesity and indicate a potential role for physical literacy programmes in schools. Whitehead (2010) [89] placed special emphasis on physical literacy being defined by competence-based and interest-based motivation in PE [89]. Chen (2015) recently suggested that a physically literate person should be characterised by self-regulated motivation for physical activity [90]. For adolescents with obesity, the PE experiences should be educational, including learning the values and benefits of physical activity for health and quality of life for sustained participation in physical activity. Regular aerobic physical activity, as part of the school curriculum or extracurricular, was shown to benefit children’s and adolescents’ cognition and academic achievement [18, 91]. Children and adolescents with obesity seem to be even more responsive to physical activity programmes for improved cognitive abilities and academic achievement [22, 92•]. This demands PE teachers more to fully understand the complexity of motivation processes in order to deliver positive PE experiences for adolescents with obesity. Furthermore, school-wide policy action is needed to address weight-based teasing/bullying.

## Conclusion

The educational cost of obesity is primarily evident for adolescent girls’ maths achievement potentially mediated by psychosocial distress and lower executive cognitive functions. There is less consistent evidence for other academic subjects though, suggesting differential relationships. The high prevalence of obesity in adolescent girls means that in addition to the threat to physical and mental health, this large population group is at risk of poor educational outcomes. Poor academic achievement might have long-term consequences on later life opportunities and economic implications. Therefore, findings of this review provide developmental and economic arguments for improved efforts in promoting psychosocial well-being and cognitive abilities linked to academic achievement in adolescent girls with obesity.
